# Thymoquinone Upregulates microRNA-199a-3p and Downregulates COX-2 Expression and PGE_2_ Production via Deactivation of p38/ERK/JNK-MAPKs and p65/p50-NF-κB Signaling in Human Lung Cancer Cells

**DOI:** 10.3390/biology14101348

**Published:** 2025-10-02

**Authors:** Yusuf Saleem Khan, Aisha Farhana, Ghorashy E. Y. Mohammed, Abuzar Abdulwahab Osman, Abdullah Alsrhani, Syed M. A. Shahid, Mohammed Kuddus, Zafar Rasheed

**Affiliations:** 1 Department of Anatomy, College of Medicine, University of Hail, Hail 55476, Saudi Arabia; y.salem@uoh.edu.sa (Y.S.K.); 2 Department of Clinical Laboratory Sciences, College of Applied Medical Sciences, Jouf University, Sakaka 72388, Saudi Arabia; afarhana@ju.edu.sa (A.F.); afalserhani@ju.edu.sa (A.A.); 3 Department of Pathology, College of Medicine, University of Hail, Hail 55476, Saudi Arabia; g.eltaybe@uoh.edu.sa (G.E.Y.M.); 4 Department of Pharmacology, College of Medicine, University of Hail, Hail 55476, Saudi Arabia; a.osman@uoh.edu.sa (A.A.O.); 5 Department of Biochemistry, College of Medicine, University of Hail, Hail 55476, Saudi Arabia; sm.shahid@uoh.edu.sa (S.M.A.S.); mkuddus@gmail.com (M.K.); 6 Department of Pathology, College of Medicine, Qassim University, P.O. Box 6655, Buraidah 51452, Saudi Arabia; zafarrasheed@qu.edu.sa (Z.R.)

**Keywords:** thymoquinone, lung cancer, microRNA-199a-3p, MAPK pathway, NF-κB signaling, COX-2, PGE_2_, anti-inflammatory

## Abstract

Chronic inflammation driven by Cyclooxygenase-2 (COX-2) overexpression plays a key role in lung cancer (LC) progression, making it a critical therapeutic target. This study explores thymoquinone (TQ), a potent bioactive phytochemical derived from Nigella sativa, known for its anti-inflammatory and anti-cancer effects, focusing on its ability to suppress lipopolysaccharide (LPS)-induced COX-2 expression via microRNA hsa-miR-199a-3p modulation in LC cells. Using A549 and SHP-77 LC cells, we tested the effect of TQ under LPS stimulation and miRNA inhibition. Advanced techniques like TaqMan qPCR, luciferase reporter gene constructs, and anti-miRNA transfection confirmed that miR-199a-3p directly silences COX-2. Western blot and ELISA assays revealed that TQ dramatically reduces COX-2 protein and PGE2 levels by boosting miRNA-199a-3p. Importantly, TQ also blocked MAPK (p38, JNK, ERK) and NF-κB activation, even when miR-199a-3p was suppressed, proving its multi-targeted action beyond miRNA regulation. These findings reveal a novel anti-inflammatory mechanism, where TQ curbs COX-2-driven inflammation by enhancing miR-199a-3p, simultaneously shutting down pro-cancer MAPK/NF-κB signaling pathways. Given the strong link between chronic inflammation and LC aggressiveness, this study positions TQ as a promising therapeutic candidate, especially for inflammation-mediated lung cancer progression. Its dual ability to modulate miRNA and key signaling cascades makes it a compelling option for future LC treatment strategies.

## Simple Summary

This study investigates thymoquinone (TQ), a natural compound from *Nigella sativa*, and its ability to reduce inflammation in lung cancer (LC) by targeting COX-2, a key enzyme in cancer progression. TQ was found to suppress COX-2 expression by increasing levels of the microRNA miR-199a-3p, which silences COX-2. Experiments in LC cells showed that TQ also inhibits pro-inflammatory MAPK and NF-κB signaling pathways, even when miR-199a-3p is blocked. These results highlight TQ’s dual anti-inflammatory and anti-cancer effects, making it a promising therapeutic option for inflammation-driven lung cancer.

## 1. Introduction

Lung cancer (LC) stands as the leading cause of cancer-related deaths worldwide, surpassing the combined mortality rates of prostate, breast, and colorectal cancers [1]. Despite the availability of surgical, chemotherapeutic, and radiotherapeutic options, overall survival remains poor, with a five-year survival rate of approximately 15% [2]. Even when detected at an early stage and treated surgically, long-term outcomes remain suboptimal. Many patients eventually experience disease recurrence, often due to undetected distant micrometastases, suggesting that occult metastasis is present even in apparently localized tumors [1,2]. These high-risk patients may benefit from adjuvant therapy; however, the inability to accurately identify them has limited the success of such interventions [3]. As a result, many clinicians have adopted a conservative approach in the absence of predictive markers for therapeutic response.

The identification of molecular and genetic biomarkers holds promise for stratifying patients into clinically relevant subgroups, potentially improving treatment outcomes [4]. Such markers not only offer prognostic value but may also serve as therapeutic targets [4,5]. Among the most studied molecules are prostaglandins, cyclooxygenases (COXs), and microRNAs (miRNAs), which have been implicated in various oncogenic processes [6]. The initial observation that nonsteroidal anti-inflammatory drugs (NSAIDs) reduce polyp formation in patients with familial adenomatous polyposis spurred interest in the role of COX enzymes in cancer [7]. Specifically, the inducible isoform COX-2 (prostaglandin-endoperoxide synthase 2) has been found to be upregulated in several tumors and is associated with inflammation-driven tumorigenesis and metastasis [8].

In parallel, miRNAs, small non-coding RNAs that regulate gene expression, have gained attention for their role in cancer biology. Several miRNAs, including those targeting COX-2, are known to modulate oncogenic and inflammatory pathways, presenting a potential for therapeutic exploitation [9]. Both COX-2 and miRNAs have been implicated as contributors to poor prognosis in lung cancer, and their modulation could offer therapeutic benefit [10]. The availability of selective COX-2 inhibitors and miRNA modulators has opened new opportunities to interfere with cancer development and progression at the molecular level [11].

Thymoquinone (TQ), the major active ingredient derived from *Nigella sativa* (commonly known as black seed), has demonstrated a wide range of biological activities, including anti-inflammatory, antioxidant, and anti-cancer effects [12,13,14]. While its anti-cancer mechanisms have been investigated in several models, its precise impact on miRNA-mediated regulation of COX-2 expression and PGE2 synthesis in lung cancer remains unclear. Prior research has shown that hsa-miR-199a-3p (miR-199a-3p) can negatively regulate COX-2 expression in various malignancies [15,16,17]. MicroRNA-199a-3p has emerged as a significant tumor suppressor in lung cancer. Its expression is markedly decreased in LC tissues and cell lines compared to paired non-tumor controls, with this downregulation correlated to advanced tumor stage, larger tumor size, and poorer patient outcomes [18]. Among the various microRNAs reported to regulate COX-2, miR-199a-3p stands out due to its demonstrated capacity to modulate COX-2 expression and PGE_2_ production in inflammatory models. For instance, in human osteoarthritic (OA) chondrocytes, treatment with epigallocatechin-3-O-gallate (EGCG) upregulated miR-199a-3p levels and significantly suppressed IL-1β–induced COX-2 expression and PGE_2_ production. Importantly, inhibition of miR-199a-3p reversed these effects, confirming its direct role in COX-2 regulation [16,17]. Notably, miR-199a-3p has also been shown to directly repress COX-2 protein translation in human myometrial cells. During inflammatory processes, expression of miR-199a-3p and its cluster counterpart miR-214 inversely correlates with COX-2 protein levels, and forced expression of miR-199a-3p significantly reduced COX-2 protein without affecting its mRNA, indicating a post-transcriptional regulatory mechanism [18]. Beyond its role in COX-2 regulation, miR-199a-3p exerts tangible tumor-suppressive effects in lung cancer. It is frequently downregulated in lung cancer tissues and cell lines, and its overexpression suppresses proliferation and migration while inducing apoptosis in lung cancer models [19]. Thus, miR-199a-3p was chosen for this study because it uniquely intersects both inflammatory regulation of COX-2 and antitumor activity in lung cancer cells, positioning it as a compelling candidate to mediate thymoquinone’s effects on COX-2/PGE_2_ and oncogenic signaling pathways. Functional studies further demonstrate that the overexpression of miR-199a-3p significantly suppresses proliferation and migration while promoting apoptosis in LC cell lines including A549; notably, combined overexpression with miR-199a-5p enhances these anti-proliferative effects [18]. Mechanistically, miR-199a-3p targets and downregulates several inflammatory mediators and cancer-associated signaling events, which are critical for cell growth and survival [18,19,20,21]. In addition, tumor xenograft models using A549 cells carrying increased levels of miR-199a-3p show reduced tumor growth and stem-like properties, accompanied by elevated apoptosis and impaired mitochondrial function through targeting of the transcription factor ZNF217 [22]. Beyond its roles in LC, miR-199a-3p has been implicated in the regulation of cancer-related pathways across diverse cancer types. It directly targets Caveolin-2 to modulate proliferation and survival in breast and endothelial cells [23]. It also influences pathways associated with inflammation-related signaling in various tumors [24]. Taken together, these findings position miR-199a-3p as a multifunctional tumor suppressor in lung cancer, exerting anti-oncogenic effects by targeting key nodes in proliferation, stemness, apoptosis, and survival pathways. These characteristics make it an attractive focus for elucidating the molecular mechanisms by which therapeutic agents such as thymoquinone may exert anti-inflammatory and anti-cancer effects via miRNA-mediated regulation.

In this study we hypothesized that thymoquinone suppresses LPS-induced COX-2 expression and PGE_2_ production in human lung cancer cells by upregulating microRNA-199a-3p, leading to inhibition of MAPK (p38, ERK, JNK) and NF-κB (p65/p50) signaling pathways. To test this hypothesis, we pretreated well-established human lung cancer cell lines with TQ, specific MAPK inhibitors, or an NF-κB inhibitor, followed by LPS stimulation, and subsequently measured the levels of hsa-miR-199a-3p, COX-2, and PGE_2_. To further determine the role of miR-199a-3p in TQ-mediated anti-inflammatory effects, cells were transfected with either anti-miR-199a-3p (inhibitor) or pre-miR-199a-3p (mimic) and then treated with TQ, MAPK inhibitors or the NF-κB inhibitor. In these transfected cells, the levels of hsa-miR-199a-3p, COX-2, and PGE_2_ were quantified. Our findings demonstrate that TQ upregulates miR-199a-3p, leading to suppression of COX-2 expression and PGE_2_ production, together with inhibition of MAPK and NF-κB signaling in A549 and SHP-77 lung cancer cells. These results underscore TQ’s therapeutic potential as a multi-targeted agent for lung cancer treatment and provide a strong basis for future translational research.

## 2. Methods

### 2.1. Cell Culture and Treatment Protocols

Human A549 lung carcinoma cells, SHP-77 sourced from the American Type Culture Collection (Rockville, MD, USA), were cultured in RPMI 1640 medium (Sigma-Aldrich, St. Louis, MO, USA) enriched with 10% fetal bovine serum (FBS; Sigma-Aldrich Chemie GmbH, Schnelldorf, Germany) and 1% penicillin–streptomycin and maintained at 37 °C in a humidified incubator with 5% CO_2_, as previously described [25]. For experiments, cells were allowed to reach 70–80% confluency and serum-starved overnight. Cell viability was evaluated using the Cell Titer-Glo Luminescent Assay (Promega, WI, USA) after treatment with varying doses of thymoquinone (TQ; Sigma-Aldrich). For signaling pathway investigations, cells were pre-incubated with TQ (50–100 nM) or for 2 h before stimulation with lipopolysaccharide (LPS; 1.0 µg/mL, Sigma-Aldrich). Untreated cells were used as negative controls. For experiments involving MAPK and NF-κB signaling inhibitors, 2 × 10^5^ cells per well were seeded in 6-well plates and cultured until they reached 60–70% confluency. Cells were then pretreated with the specific inhibitors—ERK inhibitor PD98059 (20 µM; Cat. #9900; Cell Signaling Technology, Danvers, MA, USA), JNK inhibitor SP600125 (10 µM; Cat. #8177; Cell Signaling Technology), or p38 inhibitor SB202190 (10 µM; Cat. #559389; Calbiochem/Merck Millipore, Darmstadt, Germany)—for 2 h prior to stimulation with LPS.

### 2.2. MicroRNA Transfection and Gene Expression Analysis

For transfection experiments, A549 and SHP-77 lung cancer cells were seeded at a density of 2 × 10^5^ cells per well in 6-well plates and allowed to reach approximately 60–70% confluency. Cells were transfected with either anti-miR-199a-3p inhibitor (final concentration: 100 nM) or pre-miR-199a-3p mimic (final concentration: 100 nM) using HiPerfect Transfection Reagent (Qiagen, Hilden, Germany) at a 1:2 (µg:µL) ratio of oligonucleotide to transfection reagent, according to the manufacturer’s protocol. The oligonucleotides were diluted in Opti-MEM^®^ Reduced Serum Medium (Gibco, Billings, MT, USA) before complex formation with the transfection reagent. After 6 h of transfection, the medium was replaced with complete RPMI-1640 supplemented with 10% FBS, and cells were incubated for the indicated time points before further experiments. Transfection efficiency was evaluated by performing parallel control transfections using a Cy3-labeled negative control miRNA (Thermo Fisher Scientific, Waltham, MA, USA) at the same concentration and reagent ratio as the experimental oligonucleotides. After 24 h, cells were examined under a fluorescence microscope (Olympus IX73, Tokyo, Japan) to detect Cy3 fluorescence. Efficiency was quantified by counting Cy3-positive cells in at least five randomly selected fields per well and expressing this as a percentage of the total number of cells (as determined by DAPI nuclear staining). In our experiments, the transfection efficiency consistently exceeded 80% in both A549 and SHP-77 cell lines. After 72 h, transfected cells were treated with TQ for 2 h or stimulated with LPS for 12–24 h, following prior protocols [26]. Total RNA, including small RNAs, was isolated using the mirVana miRNA Isolation Kit (Ambion, Austin, TX, USA). cDNA synthesis was carried out with the Superscript First-Strand Kit (Applied Biosystems, Waltham, MA, USA). All microRNA-specific primers, including those for hsa-miR-199a-3p and the endogenous control RNU6B, were purchased from Life Technologies (Thermo Fisher Scientific, USA) and used according to the manufacturer’s protocol. The mRNA primers for COX-2 (PTGS2) and GAPDH were synthesized by Integrated DNA Technologies (IDT, Coralville, IA, USA). The sequences for mRNA primers were: COX-2 (PTGS2, NM_000963; Forward: 5′-CAAATCCTTGCTGTTCCCACCCAT-3′; Reverse: 5′-GTGCACTGTGTTTGGAGTGGGTTT-3′); GAPDH (NM_002046; Forward: 5′-TCGACAGTCAGCCGCATCTTCTTT-3; Reverse: 5′-ACCAAATCCGTTGACTCCGACCTT-3′. Quantitative PCR (qPCR) was performed using TaqMan assays on a StepOne Real-Time PCR System (Life Technologies, Carlsbad, CA, USA). Relative expression was calculated using the comparative ΔΔCT method [27].

### 2.3. Luciferase Reporter Assays

To validate direct targeting of COX-2 by miR-199a-3p, wild-type and mutant COX-2 3′UTR fragments were cloned into pmirGLO vectors (Promega, Madison, WI, USA) using site-directed mutagenesis kits (Agilent, Santa Clara, CA, USA). A549 cells were co-transfected with the luciferase constructs and either anti-miR-199a-3p or negative controls. For the luciferase reporter assay, 1 × 10^5^ cells (A549 or SHP-77) were seeded per well in 24-well plates and allowed to reach approximately 70% confluency before transfection. Reporter plasmids, miRNA mimics or inhibitors, and the Renilla luciferase control plasmid were co-transfected using Lipofectamine^®^ 3000 (Invitrogen, Waltham, MA, USA) following the manufacturer’s protocol. After 48 h, luciferase activity was measured using the Dual-Luciferase^®^ Reporter Assay System (Promega, USA), and firefly luciferase readings were normalized to Renilla luciferase activity following previously established methods [26].

### 2.4. Cell Transfection and Signaling Inhibitor Experiments

Cells were transfected with anti-miRNAs (100 nM) or pre-miRNAs (100 nM) using HiPerfect, and, after 72 h, they were treated with pathway-specific inhibitors targeting MAPKs or NF-κB. For these experiments, 1 × 10^5^ transfected cells per well were cultured until they reached 60–70% confluency. Transfected cells were then pretreated with the specific inhibitors—ERK inhibitor PD98059 (20 µM), JNK inhibitor SP600125 (10 µM), or p38 inhibitor SB202190 (10 µM) for 2 h. Subsequently, total RNA and protein were harvested for downstream analysis.

### 2.5. Nuclear Protein Isolation and NF-κB Activity Detection

To assess the effects of TQ and miR-199a-3p on NF-κB activation, A549 cells were transfected with either anti-miR-199a-3p or miR-NC and treated with LPS (1.0 µg/mL) for 30 min. Following PBS washes, cells were pelleted at 1500× *g* for 5 min at 4 °C, and nuclear extracts were prepared using standard methods [28]. Equivalent protein amounts were used in Transcription Factor ELISA assays (Abcam, #ab133128, Cambridge, UK) to quantify p65 and p50 subunit activity.

### 2.6. Western Blot Analysis

Protein expression of COX-2 was evaluated by SDS-PAGE followed by immunoblotting. Proteins were transferred to PVDF membranes (Millipore, Burlington, MA, USA) and probed with COX-2-specific antibodies (rabbit monoclonal, Cat. #12282; 1:1000 dilution. Cell Signaling Technology, Danvers, MA, USA) and the expression levels were compared with β-actin antibodies (mouse monoclonal, Cat. #3700; 1:2000 dilution; Cell Signaling Technology, Danvers, MA, USA). Band intensities were visualized and quantified using UNSCAN-IT software, version 7.0 (Silk Scientific, Provo, UT, USA) [16]. Original western blots are provided as Supplementary Material).

### 2.7. Prostaglandin E2 Quantification

PGE2 levels in culture supernatants were measured using an ELISA kit specific for prostaglandin E2 (R&D Systems, Minneapolis, MN, USA), according to the manufacturer’s protocols.

### 2.8. Statistical Analysis

Unless otherwise stated, all experiments were performed in three independent biological replicates (n = 3), each with three technical replicates per condition. Data are presented as mean ± standard deviation (SD) unless stated otherwise. Statistical differences among groups were analyzed using one-way ANOVA followed by Tukey’s post hoc test, or two-way ANOVA with Bonferroni correction, as appropriate. GraphPad Prism 5 (GraphPad Software, San Diego, CA, USA) was used for data analysis, and results were considered statistically significant at *p* < 0.05.

## 3. Results

### 3.1. Inverse Correlation Between hsa-miR-199a-3p Expression and COX-2/PGE2 Induction in LPS-Stimulated A549 Lung Cancer Cells

**Figure 1:**
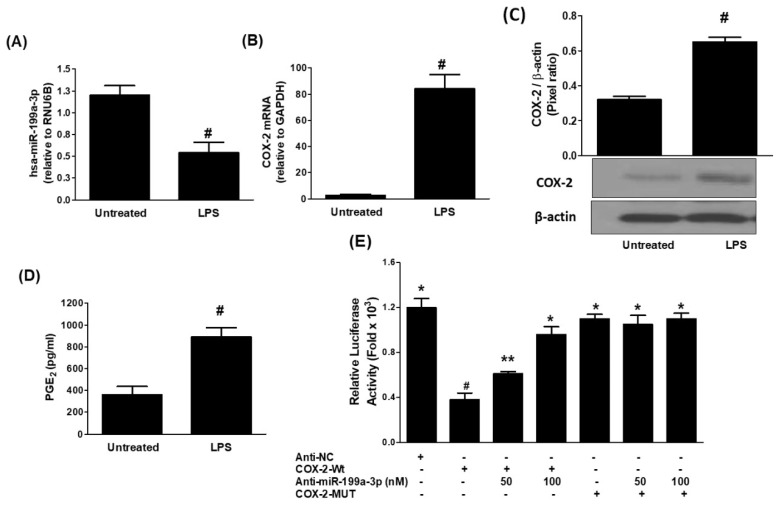
LPS downregulates hsa-miR-199a-3p and upregulates COX-2 expression in lung cancer cells. (**A**) Expression of microRNA-199a-3p in A549 cells (3 × 10^6^ cells/mL) treated with 1.0 µg/mL LPS for 24 h, measured by specific TaqMan assays. Untreated cells served as controls; RNU6B was used as an endogenous reference. Data represent mean ± SD of three independent experiments (# *p* < 0.001 vs. untreated). (**B**) COX-2 mRNA levels in A549 cells treated with 1 µg/mL of LPS for 24 h, assessed by TaqMan assays with GAPDH as endogenous control. Data are mean ± SD from three independent experiments (# *p* < 0.001 vs. untreated). (**C**) COX-2 protein expression in A549 cells treated with 1 µg/mL of LPS for 24 h, analyzed by Western blot. β-actin was used as a loading control. Band intensities were quantified using Un-Scan-It software and normalized to β-actin. Data represent mean ± SD of three independent experiments (# *p* < 0.01 vs. untreated). (**D**) PGE2 levels in culture medium of A549 cells treated with 1 µg/mL of LPS for 24 h, measured by ELISA. Data represent mean ± SD of three independent experiments (# *p* < 0.001 vs. untreated). (**E**) Luciferase reporter activity in A549 cells transfected with COX-2 wild-type (Wt) 3′UTR reporter vector and anti-miR-199a-3p. Controls included anti-NC (negative control), COX-2 mutant (MUT, negative control), and COX-2 Wt (positive control). Data show mean ± SD of three independent experiments (* *p* < 0.001 vs. #; # *p* < 0.05 vs. **; ** *p* < 0.05 vs. *).

To explore the relationship between hsa-miR-199a-3p and COX-2 expression in lung cancer cells, A549 cells were treated with 1.0 µg/mL of LPS for 24 h. This stimulation led to a marked suppression in hsa-miR-199a-3p expression (*p* < 0.05; Figure 1A). Conversely, COX-2 mRNA expression was significantly upregulated under the same conditions (*p* < 0.0001; Figure 1B). These mRNA findings were corroborated at the protein level, where Western blot analysis showed a substantial increase in COX-2 protein in LPS-treated cells compared to untreated controls (*p* < 0.01; Figure 1C). Given COX-2’s role in driving prostaglandin E2 (PGE2) biosynthesis, PGE2 levels in the culture medium were also assessed. As expected, LPS stimulation significantly elevated extracellular PGE2 concentrations (Figure 1D). To determine whether COX-2 upregulation is directly linked to hsa-miR-199a-3p suppression, luciferase reporter assays were conducted using A549 cells co-transfected with COX-2 wild-type 3′UTR and anti-miR-199a. This co-transfection led to a concentration-dependent increase in luciferase activity compared to cells receiving the COX-2-Wt vector alone (*p* < 0.05). Cells transfected with COX-2-Wt exhibited significantly reduced luciferase activity compared to those with COX-2-Mut or anti-miR control vectors (*p* < 0.05), confirming the specific binding of hsa-miR-199a-3p to the 3′UTR of COX-2 mRNA (Figure 1E).

### 3.2. Thymoquinone Reverses LPS-Induced COX-2 and PGE2 Upregulation via hsa-miR-199a-3p Induction

**Figure 2:**
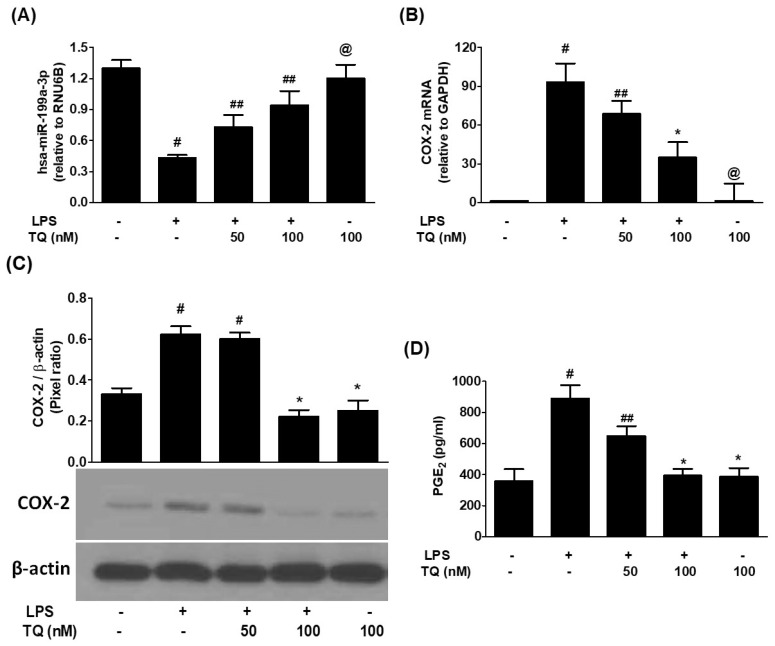
TQ upregulates hsa-miR-199a-3p and reduces COX-2 expression in LPS-treated lung cancer cells. (**A**) Expression of hsa-miR-199a-3p measured by TaqMan assays; RNU6B used as endogenous control. A549 cells (3 × 10^6^ cells/mL) pretreated with 50–100 nM TQ for 2 h, then stimulated with LPS for 24 h. Data represent mean ± SD of three experiments (# *p* < 0.01 vs. untreated; # *p* < 0.05 vs. ##; @ *p* < 0.05 vs. # or ##). (**B**) COX-2 mRNA levels measured by TaqMan assays with GAPDH as endogenous control in similarly treated cells. Data are mean ± SD of three experiments (# *p* < 0.0001 vs. untreated; # *p* < 0.05 vs. ##; # *p* < 0.05 vs. *; * *p* < 0.05 vs. ##; @ *p* < 0.01 vs. #). (**C**) COX-2 protein levels measured by Western blot, normalized to β-actin. Data represent mean ± SD of three independent experiments (# *p* < 0.05 vs. untreated; # *p* < 0.05 vs. *). (**D**) PGE2 production measured by ELISA in culture medium of TQ-pretreated and LPS-stimulated A549 cells. Data represent mean ± SD of three independent experiments (# *p* < 0.001 vs. untreated; # *p* < 0.05 vs. ##; # *p* < 0.05 vs. *; * *p* < 0.05 vs. ##).

To assess the regulatory influence of thymoquinone (TQ), A549 cells were pretreated with TQ (50–100 nM) before LPS exposure. TQ significantly enhanced the expression of hsa-miR-199a-3p in a dose-dependent manner (*p* < 0.05; Figure 2A). This upregulation was accompanied by a marked decline in both COX-2 mRNA and protein levels (*p* < 0.05; Figure 2B,C), demonstrating TQ’s suppressive effects on COX-2 expression. Correspondingly, TQ pretreatment reduced PGE2 levels in the conditioned media of LPS-stimulated cells (*p* < 0.05; Figure 2D).

### 3.3. Thymoquinone-Mediated Suppression of COX-2 Is Dependent on hsa-miR-199a-3p Activity

**Figure 3:**
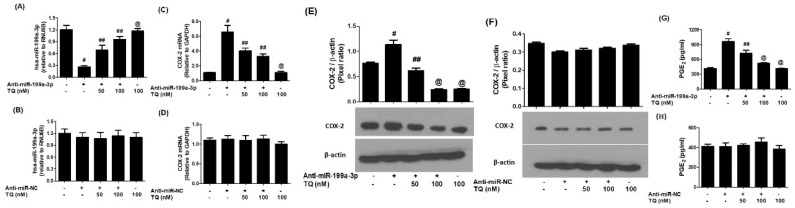
TQ upregulates hsa-miR-199a-3p and reduces COX-2 expression in lung cancer cells transfected with anti-miR-199a-3p. (**A**) hsa-miR-199a-3p levels measured by TaqMan assays; RNU6B used as endogenous control. A549 cells (3 × 10^6^ cells/mL) transfected with anti-miR-199a-3p and treated with 50–100 nM TQ for 24 h. Data represent mean ± SD of three experiments (# *p* < 0.01 vs. untreated; # *p* < 0.05 vs. ##; @ *p* < 0.05 vs. #). (**B**) hsa-miR-199a-3p expression in A549 cells transfected with anti-miR-NC and treated with TQ, measured by TaqMan assays. Data represent mean ± SD of three experiments. (**C**) COX-2 mRNA expression in A549 cells transfected with anti-miR-199a-3p and treated with TQ for 24 h (# *p* < 0.001 vs. non-transfected; ## *p* < 0.05 vs. @; @ *p* < 0.05 vs. #). (**D**) COX-2 mRNA expression in cells transfected with anti-miR-NC and treated with TQ. (**E**) COX-2 protein expression analyzed by Western blot in cells transfected with anti-miR-199a-3p and treated with TQ; β-actin used as control. Data represent mean ± SD (# *p* < 0.001 vs. non-transfected; # *p* < 0.05 vs. ##; ## *p* < 0.05 vs. @; # *p* < 0.05 vs. @). (**F**) COX-2 protein expression in cells transfected with anti-miR-NC and treated with TQ. (**G**) PGE2 production in culture medium of cells transfected with anti-miR-199a-3p and treated with TQ, measured by ELISA. Data represent mean ± SD (# *p* < 0.001 vs. non-transfected; # *p* < 0.05 vs. ##; ## *p* < 0.05 vs. @; # *p* < 0.05 vs. @). (**H**) PGE2 production in culture medium of cells transfected with anti-miR-NC and treated with TQ.

To determine whether TQ’s suppression of COX-2 expression operates through hsa-miR-199a-3p, anti-miR-199a-3p was transfected into A549 cells. Transfection with anti-miR-199a effectively reduced endogenous hsa-miR-199a-3p levels, which were subsequently restored upon treatment with TQ in a dose-responsive fashion (Figure 3A). As a negative control, cells transfected with anti-miR-NC did not show significant changes in miR-199a-3p expression, even when treated with TQ (Figure 3B). Knockdown of hsa-miR-199a-3p led to a significant rise in COX-2 mRNA expression (*p* < 0.01; Figure 3C), which was reversed by TQ treatment (*p* < 0.05). A parallel trend was observed at the protein level, where Western blot analysis confirmed that TQ effectively suppressed COX-2 protein in anti-miR-199a-transfected cells (*p* < 0.05; Figure 3E). No such changes were observed in anti-miR-NC-transfected cells (Figure 3D,F). Furthermore, ELISA data demonstrated that TQ significantly reduced PGE2 secretion in the media of anti-miR-199a-transfected cells (*p* < 0.05; Figure 3G), while no such effects were observed in the anti-miR-NC-transfected cells (*p* > 0.05; Figure 3H).

### 3.4. MAPK Pathway Involvement in Thymoquinone-Driven hsa-miR-199a-3p Modulation and COX-2/PGE2 Suppression

**Figure 4:**
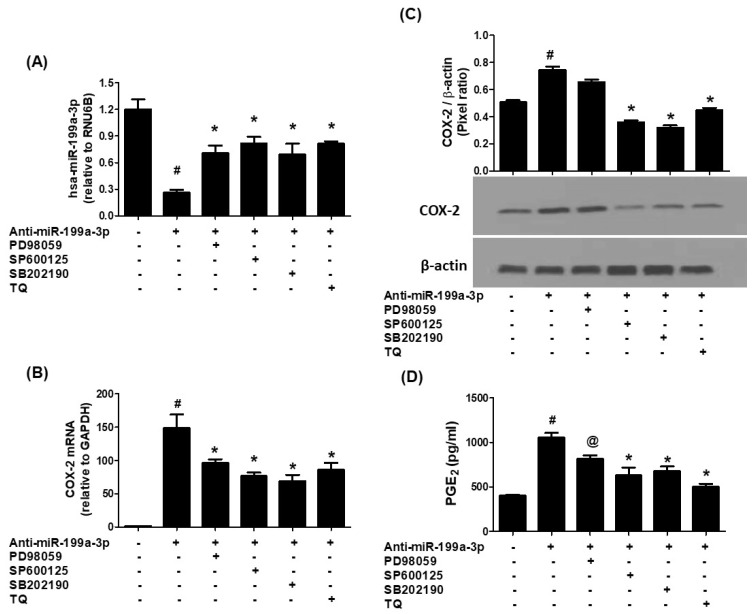
MAPK inhibitors and TQ upregulate hsa-miR-199a-3p and downregulate COX-2 expression in lung cancer cells transfected with anti-miR-199a-3p. (**A**) hsa-miR-199a-3p expression measured by TaqMan assays; RNU6B as endogenous control. A549 cells transfected with anti-miR-199a-3p and treated with ERK inhibitor (PD98059), JNK inhibitor (SP600125), p38 inhibitor (SB202190), or TQ for 24 h. Data show mean ± SD (# *p* < 0.01 vs. non-transfected; # *p* < 0.05 vs. *). (**B**) COX-2 mRNA expression in similarly treated cells. Data represent mean ± SD (# *p* < 0.0001 vs. non-transfected; * *p* < 0.05 vs. #). (**C**) COX-2 protein levels by Western blot normalized to β-actin. Data represent mean ± SD (# *p* < 0.05 vs. non-transfected; # *p* < 0.05 vs. *). (**D**) PGE2 production measured by ELISA in culture medium of transfected and treated cells. Data represent mean ± SD (# *p* < 0.001 vs. non-transfected; # *p* < 0.05 vs. @; @ *p* < 0.05 vs. *).

To elucidate whether MAPK signaling influences TQ-mediated effects, A549 cells transfected with anti-miR-199a were treated with specific MAPK inhibitors: SB202190 (p38), SP600125 (JNK), PD98059 (ERK), or TQ. Transfection led to significant downregulation of hsa-miR-199a-3p (*p* < 0.001). However, treatment with either MAPK inhibitors or TQ markedly restored miR-199a-3p expression levels (*p* < 0.01; Figure 4A). These treatments also suppressed COX-2 mRNA and protein expression in the transfected cells (Figure 4B,C, *p* < 0.05). Consistently, PGE2 production was significantly reduced upon MAPK inhibition or TQ exposure (Figure 4D), indicating that suppression of ERK, JNK, and p38 MAPK activities contributes to TQ’s regulatory effects.

### 3.5. Thymoquinone Inhibits NF-κB Activation via hsa-miR-199a-3p Induction

**Figure 5:**
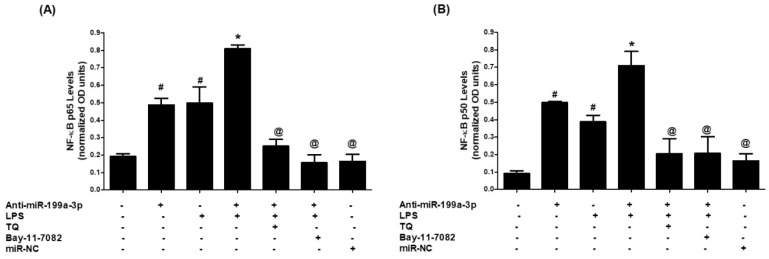
TQ inhibits LPS-induced NF-κB activation in lung cancer cells transfected with anti-miR-199a-3p. (**A**) LPS-induced NF-κB p65 activation was inhibited by TQ in A549 cells transfected with anti-miR-199a-3p. NF-κB p65 activity measured by a transcription factor assay (Abcam). Bay-11-7082 served as a selective NF-κB inhibitor control. Data show mean ± SEM from five independent experiments (# *p* < 0.01 vs. untreated or miR-NC; * *p* < 0.05 vs. #; @ *p* < 0.01 vs. # or *). (**B**) LPS-induced NF-κB p50 activation was similarly inhibited by TQ in transfected A549 cells. Data represent mean ± SEM (# *p* < 0.01 vs. untreated or miR-NC; * *p* < 0.05 vs. #; @ *p* < 0.01 vs. # or *).

Next, the involvement of NF-κB signaling in TQ-induced hsa-miR-199a-3p upregulation was explored. A549 cells transfected with anti-miR-199a-3p were pretreated with either Bay-11-7082 (NF-κB inhibitor) or TQ and then stimulated with LPS. Nuclear extracts were examined for p65 and p50 activation. Anti-miR-199a transfection increased nuclear p65 levels, further amplified by LPS stimulation (*p* < 0.001). Both Bay-11-7082 and TQ pretreatment significantly curtailed this activation (*p* < 0.001; Figure 5A). A similar trend was observed for p50 nuclear translocation, where TQ and Bay-11-7082 significantly reduced p50 levels elevated by LPS (*p* < 0.001; Figure 5B). These results suggest that TQ counters NF-κB activation by upregulating hsa-miR-199a-3p, thereby attenuating COX-2 expression.

### 3.6. Validation of Data Using an Additional Human Lung Cancer Cell Line (SHP-77)

**Figure 6:**
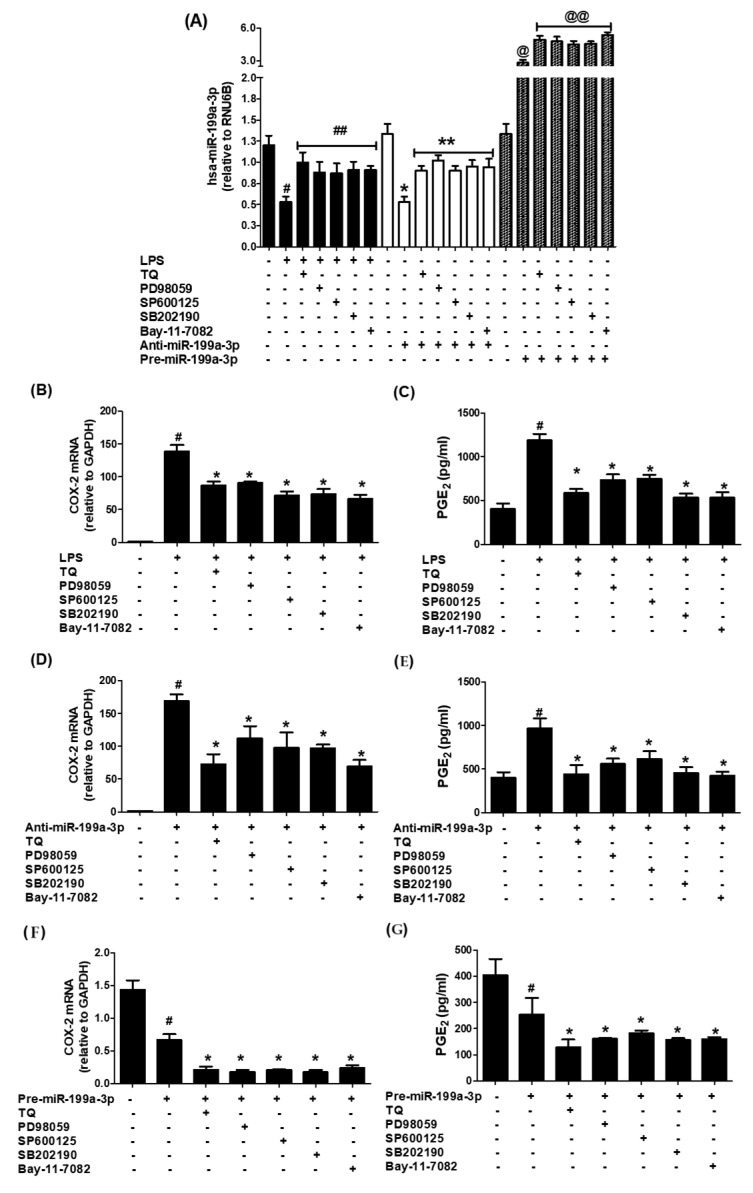
TQ, MAPK inhibitors and NF-κB inhibitors upregulate hsa-miR-199a-3p and downregulate COX-2 expression and PGE_2_ production in SHP-77 human lung cancer cells treated with LPS (**A**–**C**), transfected with anti-miR-199a-3p (**A**,**D**,**E**) and pre-anti-miR-199a-3p (**A**,**F**,**G**) expression measured by TaqMan assays and PGE_2_-specific ELISAs. SHP-77 cells were treated with LPS or transfected with anti-miR-199a-3p/pre-miR-199a-3p and treated with TQ, ERK inhibitor (PD98059), JNK inhibitor (SP600125), p38 inhibitor (SB202190), or NF-κB inhibitor (Bay-11-7082) for 24 h. Data show mean ± SD (# *p* < 0.01 vs. untreated cells; # *p* < 0.05 vs. ##), (* *p* < 0.01 vs. miR-control-transfected; * *p* < 0.05 vs. **), (@ *p* < 0.01 vs. miR-control-transfected; @ *p* < 0.05 vs. @@).

To further validate the therapeutic role of TQ, experiments were conducted using an additional human lung cancer cell line, SHP-77. Cells were pretreated with TQ (100 nM) prior to LPS exposure. LPS treatment markedly reduced hsa-miR-199a-3p expression (*p* < 0.001), whereas TQ pretreatment significantly restored its expression (*p* < 0.05; Figure 6A). This TQ-induced upregulation of hsa-miR-199a-3p was accompanied by a significant reduction in both COX-2 expression (*p* < 0.05; Figure 6B) and PGE_2_ production (*p* < 0.05; Figure 6C).

To determine the involvement of MAPK and NF-κB signaling pathways in TQ-mediated effects, SHP-77 cells were transfected with anti-miR-199a-3p and treated with specific MAPK inhibitors—SB202190 (p38), SP600125 (JNK), PD98059 (ERK)—or TQ. Anti-miR-199a-3p transfection significantly downregulated hsa-miR-199a-3p expression (*p* < 0.001; Figure 6A). Notably, treatment with each MAPK inhibitor or the NF-κB inhibitor markedly restored hsa-miR-199a-3p levels (*p* < 0.01; Figure 6A) and concurrently suppressed COX-2 expression (*p* < 0.05; Figure 6D) and PGE_2_ production (*p* < 0.05; Figure 6E).

These findings were further corroborated in SHP-77 cells transfected with pre-miR-199a-3p (mimic), which showed robust upregulation of hsa-miR-199a-3p (*p* < 0.001; Figure 6A). Subsequent treatment with TQ, MAPK inhibitors, or NF-κB inhibitor further enhanced miR-199a-3p expression (*p* < 0.05) while significantly reducing COX-2 expression (*p* < 0.05; Figure 6D) and PGE_2_ production (*p* < 0.05; Figure 6E). Taken together, these consistent results in both A549 and SHP-77 lung cancer cell lines demonstrate that inhibition of ERK, JNK, p38 MAPK, and NF-κB signaling pathways plays a pivotal role in TQ’s regulatory effects.

**Figure 7:**
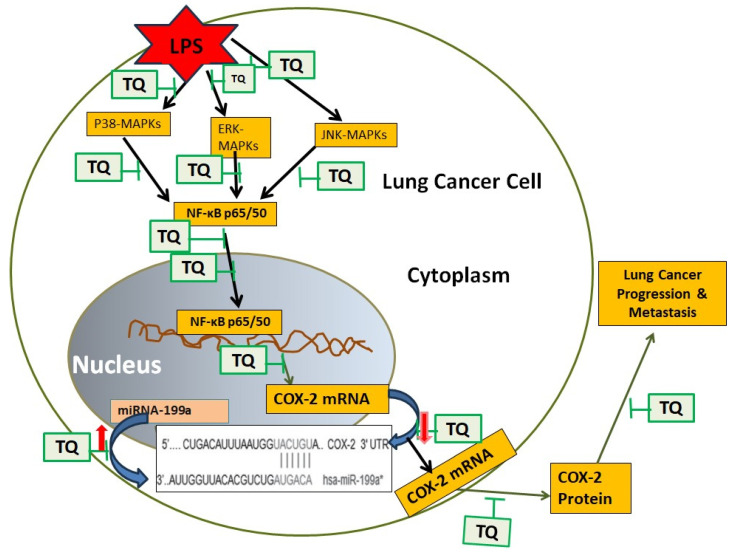
Schematic overview of thymoquinone-mediated therapeutic effects. Diagram illustrating TQ’s suppression of LPS-induced MAPKs and NF-κB signaling pathways, upregulation of hsa-miR-199a-3p (indicated in figure as hsa-miR-199a*), and consequent downregulation of COX-2 expression in lung cancer cells, highlighting its potential to inhibit metastasis and cancer progression.

Collectively, these results illustrate that LPS-induced COX-2 overexpression and PGE2 production are mediated through the suppression of hsa-miR-199a-3p via MAPK and NF-κB activation. Thymoquinone acts as a potent inhibitor of these pathways, restoring hsa-miR-199a-3p levels and thereby repressing COX-2 and PGE2. This mechanism is depicted schematically in Figure 7, highlighting TQ’s potential as a therapeutic agent against lung cancer progression.

## 4. Discussion

This study is the first to demonstrate the therapeutic potential of thymoquinone (TQ) in mitigating inflammation mediated by COX-2 and PGE_2_ by inhibiting the MAPK pathways (p38, JNK, ERK) and the NF-κB p65/p50 axis, via upregulation of hsa-miR-199a-3p in human lung cancer cells. We also acknowledge that TQ’s anti-inflammatory effects may be only partly mediated by hsa-miR-199a-3p upregulation and that additional in-depth studies, including knockout approaches, are needed to fully establish causality. COX-2 plays a pivotal role in inflammation, chiefly through its involvement in prostaglandin synthesis, and its expression is frequently heightened under inflammatory conditions [29]. While various strategies currently exist for lung cancer management, identifying novel biomarkers remains essential for enabling earlier detection and refining prognostic accuracy [30]. Although COX-2 alone may not serve as a stand-alone prognostic marker for lung cancer, several upstream and downstream effectors within the COX-2 pathway have demonstrated potential as biomarkers for predicting cancer prognosis and metastatic potential [31]. For example, elevated levels of PGE2—a primary enzymatic product of COX-2—have been linked to significantly reduced five-year survival rates in lung cancer patients, highlighting its prognostic relevance [29]. Moreover, co-expression of BPTF, a known COX-2 promoter-binding protein, with COX-2 has been associated with unfavorable clinical outcomes [30]. Similarly, overexpression of Ku80, another regulatory protein binding the COX-2 promoter, has been shown to increase COX-2 levels in lung cancer cells, correlating with poorer survival outcomes [31]. Regression analysis further confirms that heightened expressions of Ku80, mPGES, and PGE2 are statistically significant predictors of lung cancer prognosis [31,32]. Collectively, these findings emphasize that COX-2 and its downstream metabolite PGE2 are integral to lung cancer progression and metastasis and serve as promising therapeutic targets.

MicroRNA-199a-3p has emerged as a potent tumor suppressor in a range of malignancies, exerting regulatory effects on cellular proliferation, migration, apoptosis, and chemoresistance. Frequently downregulated in cancer, its re-expression has shown promising therapeutic implications [15,33,34]. In osteosarcoma, hsa-miR-199a-3p is significantly underexpressed; its restoration suppresses tumor cell growth and migration by directly targeting oncogenes such as Met, mTOR, and Stat3 [35]. Additionally, hsa-miR-199a-3p enhances sensitivity to doxorubicin by modulating CD44, a molecule strongly associated with tumor aggressiveness and drug resistance [36]. In prostate cancer, it hinders tumor proliferation and invasion through downregulation of Smad1 [15], and it is notably diminished in cancer stem cells. Reintroducing hsa-miR-199a-3p in these cells reduces tumorigenic potential by targeting CD44, c-MYC, cyclin D1 (CCND1), and EGFR [37]. Under hypoxic conditions, reduced levels of hsa-miR-199a-3p result in elevated c-Met expression and activation of MAPK and PI3K cascades, thereby advancing tumor development [38]. It also restricts tumor growth, motility, and angiogenesis by targeting molecules like VEGFA, VEGFR1/2, HGF, and MMP2 [39]. In hepatocellular carcinoma, its inhibition of the PAK4/Raf/MEK/ERK pathway contributes to its tumor-suppressive role [40]. Additionally, hsa-miR-199a-3p downregulates BRCA1, impairing DNA repair and enhancing sensitivity to cisplatin and PARP inhibitors in triple-negative breast cancer (TNBC) [23]. Paradoxically, it may support growth in some breast cancer lines by targeting caveolin-2, though caveolin-2 overexpression can reverse these effects [41]. Altogether, miR-199a-3p regulates a broad range of oncogenic pathways, positioning it as a compelling candidate for diagnostic and therapeutic applications. Despite the breadth of evidence supporting its tumor-suppressive roles, its function in lung cancer remains poorly explored. The present study addresses this gap, demonstrating for the first time that hsa-miR-199a-3p modulates COX-2 expression and PGE2 production in LPS-stimulated A549 lung cancer cells.

TQ, a bioactive constituent of *Nigella sativa*, has gained substantial interest due to its anti-inflammatory, antioxidant, and anticancer attributes [41]. To our knowledge, this is the first report to establish that TQ suppresses inflammation by inhibiting COX-2 expression and PGE2 production through hsa-miR-199a-3p upregulation in lung cancer cells. Our data clearly reveal that TQ significantly diminishes LPS-induced expression of COX-2 and its metabolite PGE2 by enhancing hsa-miR-199a-3p levels. These findings support the growing body of evidence advocating miRNA-based therapeutic strategies for cancer treatment.

Among key inflammatory mediators, the MAPK family and NF-κB signaling pathways are especially critical in lung cancer biology. MAPK signaling governs various gene expressions, including COX-2, and has been found to be a central pathway involved in inflammation [42]. These signaling cascades translate extracellular cues into intracellular responses and are often dysregulated in pathological conditions such as cancer [43]. MAPKs have a well-documented role in regulating the expression of inflammatory genes, including COX-2 and PGE2, and modulating downstream pathways that promote cancer progression [44,45]. As highly conserved serine/threonine kinases, MAPKs are involved in crucial biological functions such as apoptosis, cell growth, stress response, and differentiation [45,46]. The three major subfamilies, p38 MAPK, JNK, and ERK, are particularly involved in inflammation-driven oncogenic signaling and are being actively investigated as drug targets [47]. In this study, we investigated how TQ influences hsa-miR-199a-3p and COX-2/PGE2 expression through modulation of MAPKs. A549 cells transfected with anti-miR-199a-3p were subsequently treated with selective inhibitors for p38 (SB202190), JNK (SP600125), and ERK (PD98059), or with TQ. The outcomes unequivocally show that these MAPK pathways play significant roles in regulating COX-2 and PGE2 via miR-199a-3p, reinforcing the interplay between MAPKs and miRNA regulation in inflammation and cancer.

The NF-κB family comprises five transcription factors, including p65 and p50, which form heterodimeric complexes that bind to specific promoter sequences to regulate inflammatory and immune responses [46,48]. Over the past few decades, our understanding of NF-κB in disease pathology has expanded considerably, especially regarding its central role in cancer onset, progression, metastasis, and therapy resistance [48]. Constitutive NF-κB activity, often driven by chronic inflammation or oncogenic mutations, supports tumor cell proliferation, inhibits apoptosis, promotes angiogenesis, and facilitates epithelial–mesenchymal transition (EMT), thereby fostering metastasis [47,48]. In some contexts, NF-κB also rewires cellular metabolism and undermines immune surveillance to promote tumor survival [47]. Therefore, suppressing NF-κB activity has shown therapeutic promise, although caution is needed due to its broad physiological importance [47]. In this study, we assessed nuclear levels of NF-κB subunits p65 and p50 in A549 cells transfected with anti-miR-199a-3p. The results indicate that LPS stimulation significantly boosts NF-κBp65 and NF-κBp50 levels in anti-miR-199a-3p-transfected cells, while TQ treatment substantially reverses this effect. These findings suggest that TQ downregulates both subunits of NF-κB through hsa-miR-199a-3p. Additionally, in the same transfected cells, TQ suppressed COX-2 expression at both mRNA and protein levels and decreased PGE2 secretion, further reinforcing the role of miR-199a-3p as a mediator of TQ’s anti-inflammatory effects. Alongside our data on MAPKs, this collective evidence supports the conclusion that TQ attenuates COX-2 expression and PGE2 production by disrupting MAPK (p38, JNK, ERK) and NF-κB (p65/p50) signaling through the upregulation of hsa-miR-199a-3p in human lung cancer cells. These findings have been further validated by using an additional human lung cancer cell line (SHP-77), which has been well established to be used as a model of human lung cancer cellular-based studies [49]. Cells pretreated with TQ prior to LPS exposure showed markedly increased hsa-miR-199a-3p expression and marked decrease of COX-2 expression and PGE2 production. To further validate these findings with SHP-77 LC cells on the cellular pathways, MAPK and NF-κB, SHP-77 cells were transfected with anti-miR-199a-3p and treated with specific MAPK inhibitors—SB202190 (p38), SP600125 (JNK), PD98059 (ERK) and Bay-11-7082 (NF-κB). Treatment with each MAPK inhibitor or the NF-κB inhibitor markedly restored hsa-miR-199a-3p levels and concurrently suppressed COX-2 expression and PGE_2_ production. Furthermore, these findings have also been further re-validated by transfection of SHP-77 LC cells with pre-miR-199a-3p (mimic). The data showed that treatment with MAPK inhibitors or NF-κB inhibitors further enhanced miR-199a-3p expression and significantly reduced COX-2 expression and PGE_2_ production. These consistent findings in both A549 and SHP-77 LC cell lines demonstrate that inhibition of ERK, JNK, p38 MAPK, and NF-κB signaling pathways plays a pivotal role in TQ’s regulatory effects. While our findings provide important insights into the role of TQ in regulating COX-2/PGE_2_ expression and inflammatory signaling through hsa-miR-199a-3p in lung cancer cells, certain limitations should be acknowledged. First, our experiments were limited to in vitro models (A549 and SHP-77 cell lines), and, thus, the relevance of these findings in vivo remains to be confirmed. Second, although we demonstrated that TQ upregulates hsa-miR-199a-3p and suppresses inflammatory signaling, we did not perform knockout or CRISPR/Cas9-based gene editing experiments to conclusively establish that these effects are entirely dependent on hsa-miR-199a-3p. Therefore, additional mechanisms independent of miR-199a-3p may also contribute to the observed effects. Future studies incorporating animal models and knockout approaches will further validate and expand upon our findings, ultimately supporting the translation of TQ into clinical applications.

## 5. Conclusions

This study is the first to demonstrate that thymoquinone (TQ), a natural bioactive compound, effectively suppresses inflammatory signaling pathways involving COX-2 and microRNA-199a-3p in human lung cancer cells. The findings reveal that TQ significantly downregulates COX-2 expression at both the transcriptional and translational levels, which correspondingly reduces the production of its downstream metabolite, PGE_2_. Furthermore, this investigation establishes that TQ inhibits all three principal MAPK pathways—p38, JNK, and ERK—and also diminishes the activation of NF-κB subunits p65 and p50 by upregulating microRNA-199a-3p. Overall, TQ modulates COX-2 through the regulation of microRNA-199a-3p, positioning it as a promising candidate for future cancer therapies due to its ability to influence multiple signaling cascades involved in tumor suppression.

## Data Availability

All data and materials used in this study are available from the corresponding author and will be provided upon reasonable request.

## Supplementary Materials

The following supporting information can be downloaded at: https://www.mdpi.com/article/10.3390/biology14101348/s1, WB original images of Figure 1C, Figure 2C, Figure 3E,F and Figure 4C.
