# Supplementary material for: Thymoquinone Upregulates microRNA-199a-3p and Downregulates COX-2 Expression and PGE2 Production via Deactivation of p38/ERK/JNK-MAPKs and p65/p50-NF-κB Signaling in Human Lung Cancer Cells
Source: Biology (Basel). 2025 Oct 2;14(10):1348. doi: 10.3390/biology14101348 (PMC12561305; doi:10.3390/biology14101348)

Figure 1C

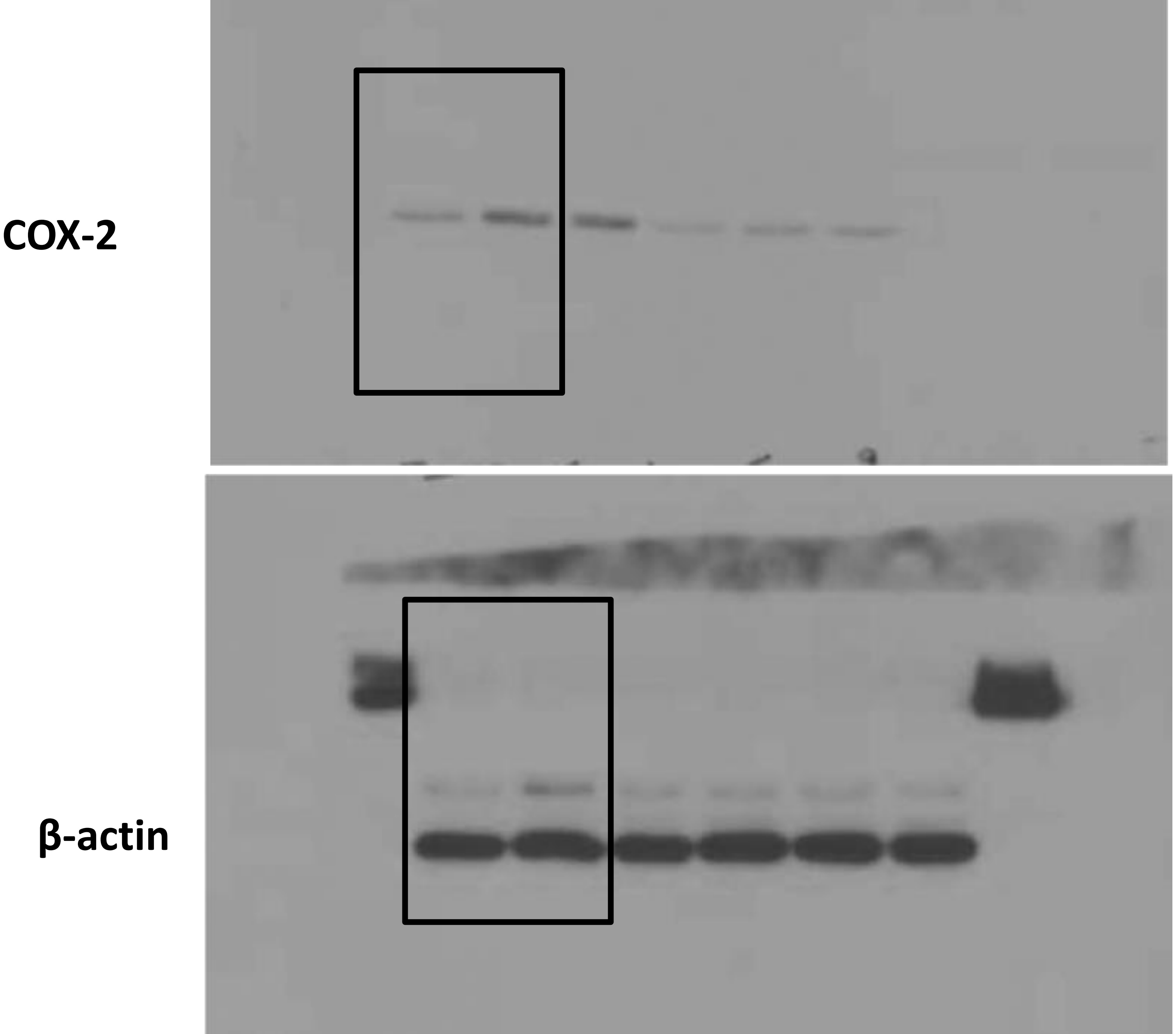

Figure 2C

COX-2

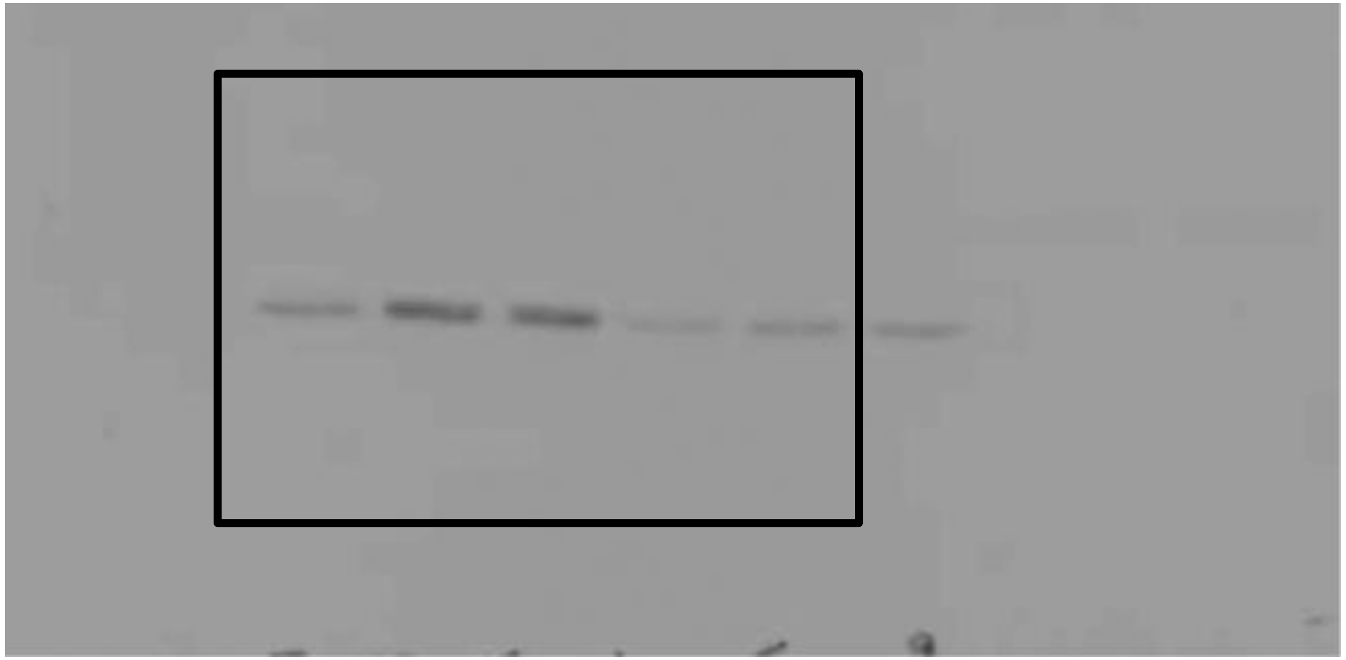

β-actin

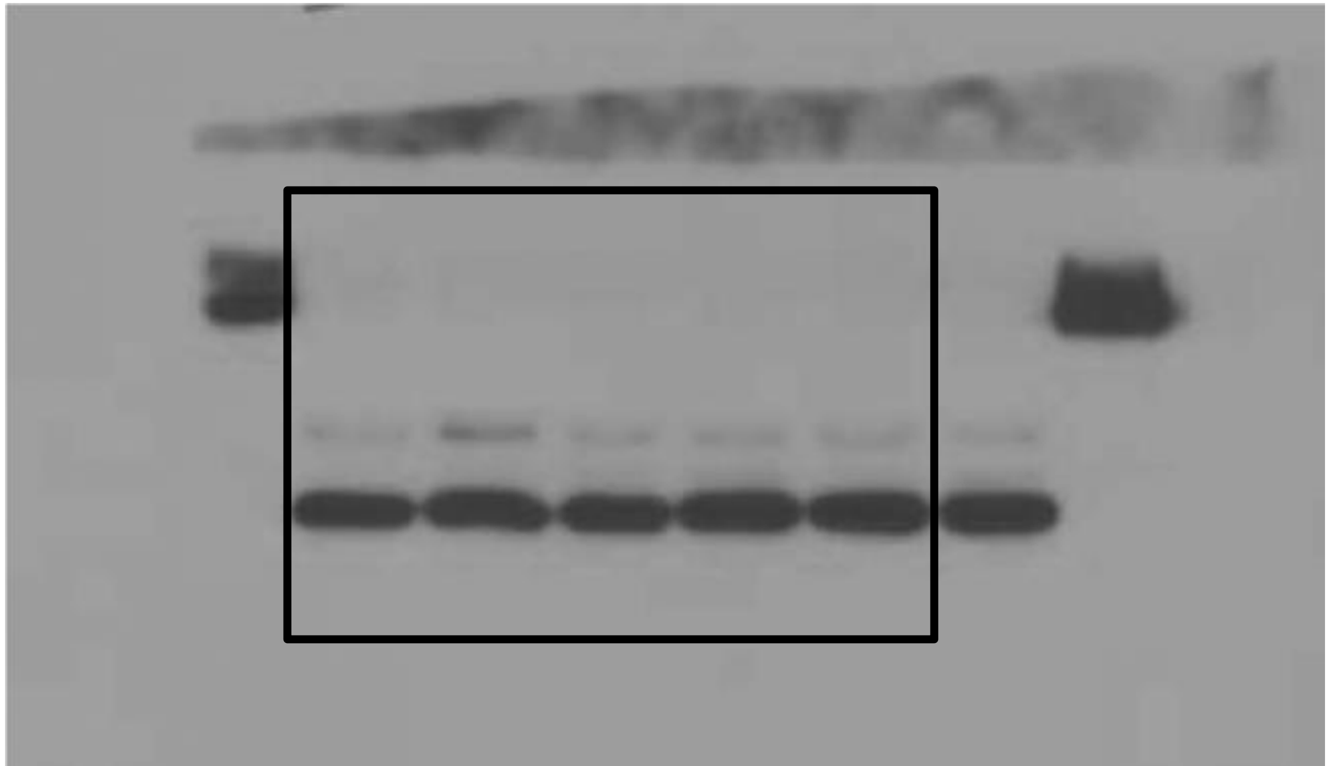

Figure 3E

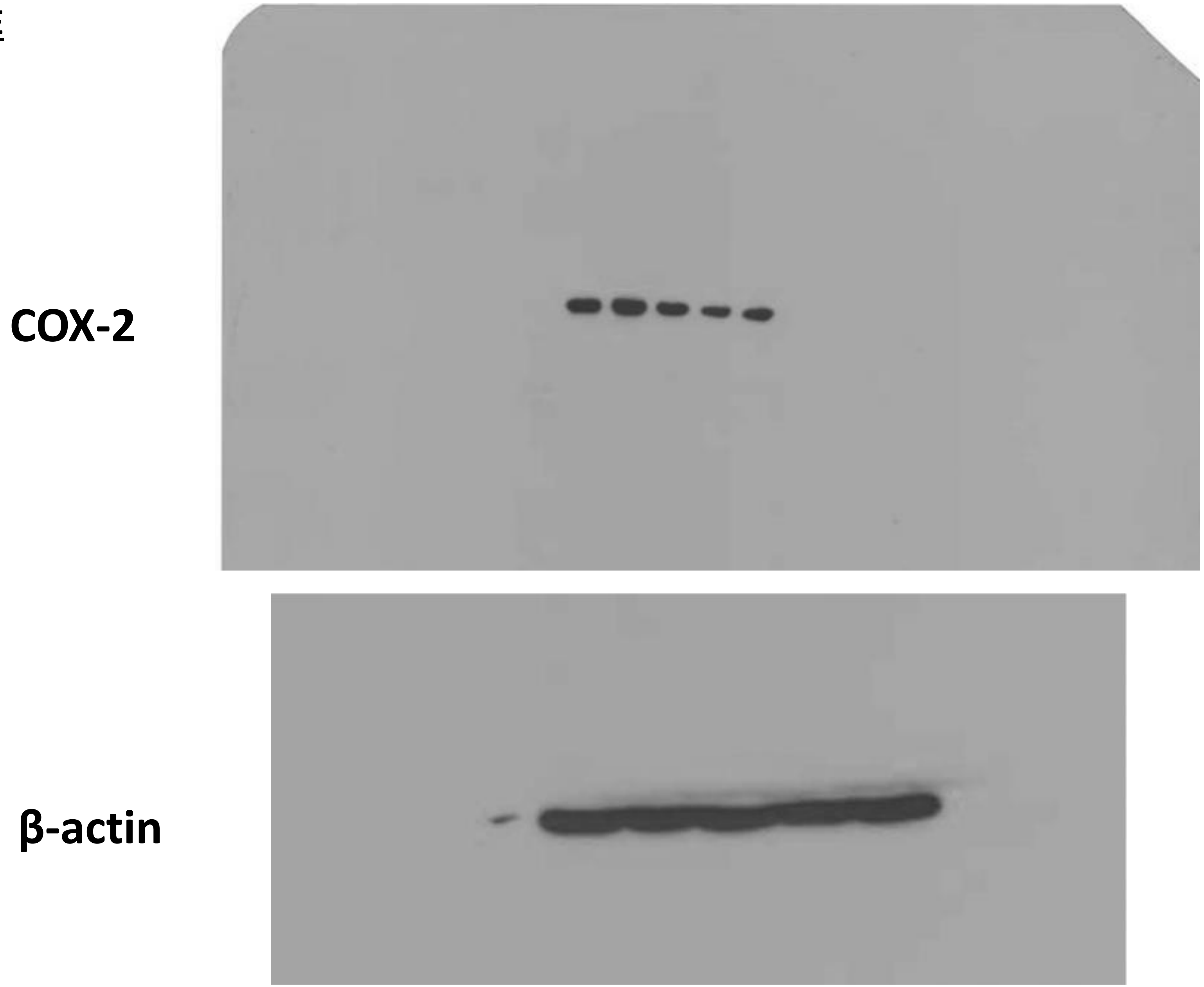

Figure 3F

COX-2

β-actin

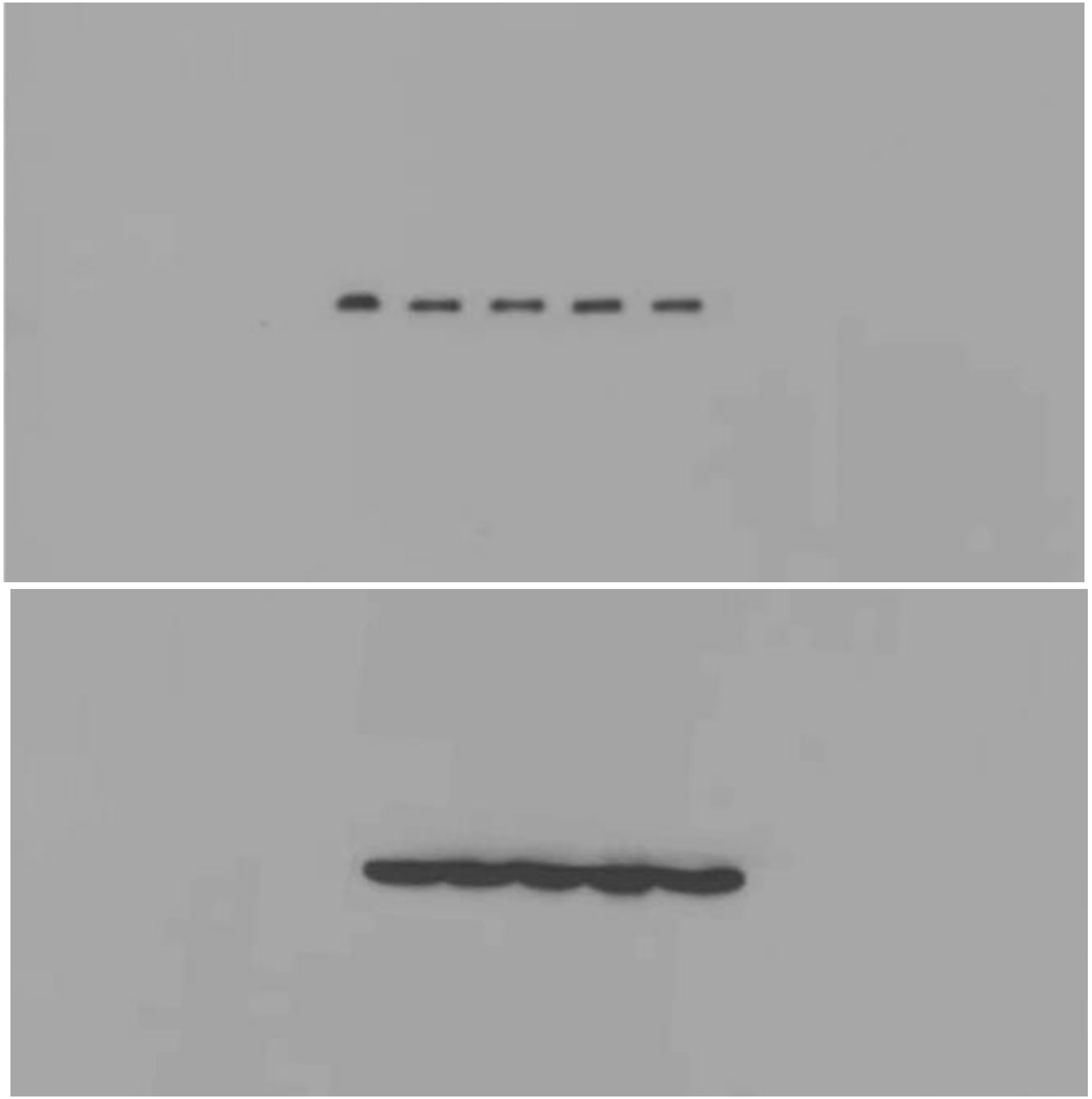

Figure 4C

COX-2

β-actin

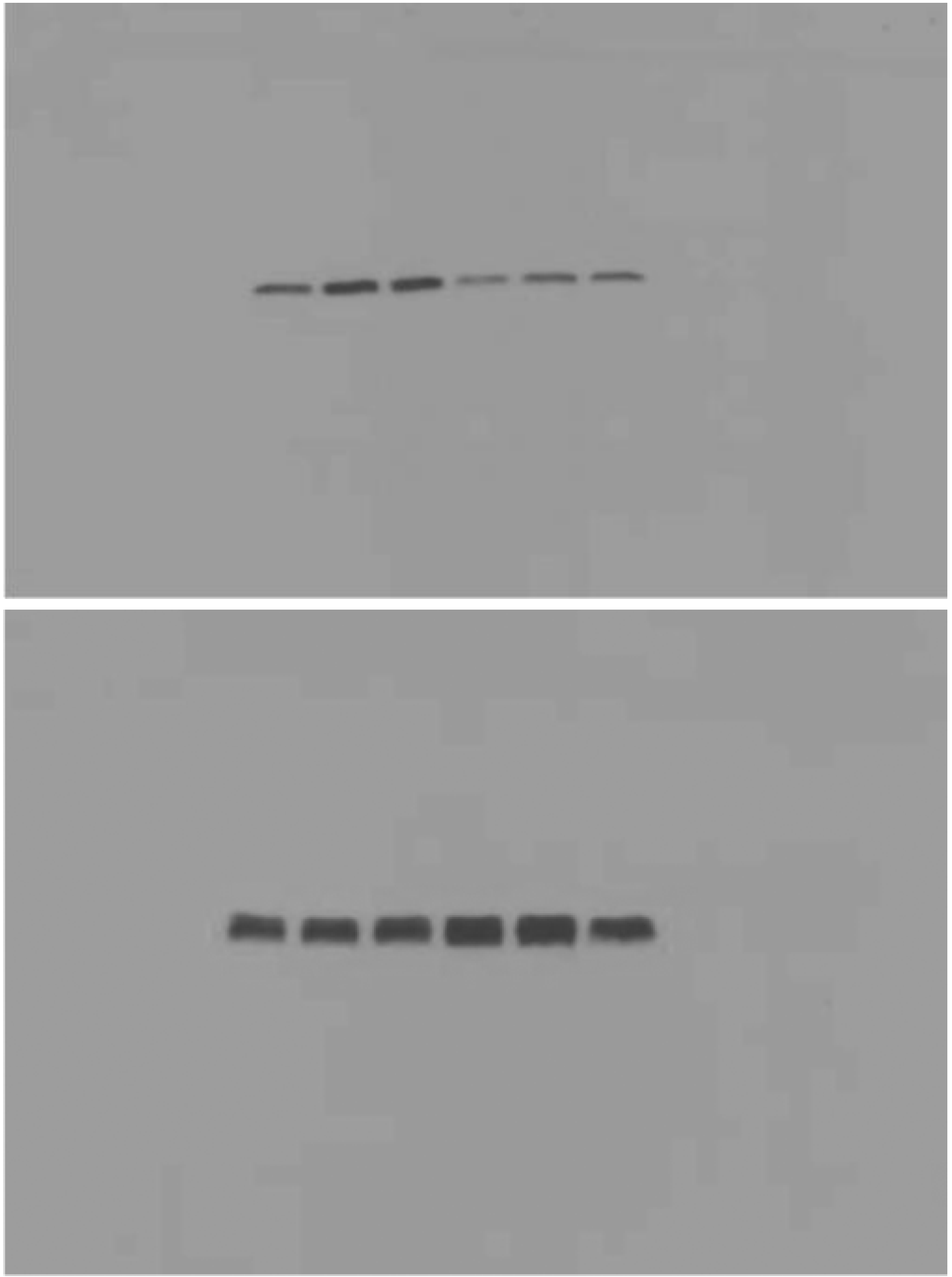

Supplement: Supplementary file 1 [file biology-14-01348-s001.zip › biology-3776591-supplementary.pdf]
